# Scleral Lenses for Managing Dry Eye Disease in the Absence of Corneal Irregularities: What Is the Current Evidence?

**DOI:** 10.3390/jcm13133838

**Published:** 2024-06-29

**Authors:** Sharon X. Qiu, Daddi Fadel, Alex Hui

**Affiliations:** 1 Centre for Ocular Research & Education, School of Optometry & Vision Science, University of Waterloo, Waterloo, ON N2L 3G1, Canada; daddi.fadel@uwaterloo.ca (D.F.); alex.hui@uwaterloo.ca (A.H.); 2 School of Optometry and Vision Science, Faculty of Medicine and Health, UNSW Sydney, Sydney, NSW 2052, Australia

**Keywords:** scleral lenses, dry eye disease, clinical decision-making, contact lenses

## Abstract

Scleral lenses (SLs) are large-diameter rigid contact lenses that are a mainstay treatment for eyes with corneal irregularities. In recent years, there has been increased interest in the role of managing dry eye disease (DED) with SLs, as many patients with DED have reported symptomatic relief with SL wear. The role of SLs for DED management when there are associated corneal irregularities is supported by individual case reports and studies. This has prompted practitioners to begin advocating using SLs in DED cases, even in the absence of associated corneal irregularities and other ocular surface diseases (OSDs). There have also been discussions on potentially placing SLs earlier in the treatment hierarchy of DED, where it currently sits at a more advanced level of intervention (Step 3) in the TFOS DEWS II Report. This review will present the currently available, albeit sparse, evidence that supports and suggests this practice, as well as ancillary evidence supporting the purported benefits of SL wear in DED. The advantages of SL wear, such as corneal healing, absence of tear evaporation and contact lens dehydration, and improved visual acuity with associated increased wear comfort, and how this will benefit DED patients will be explored. Conversely, the challenges associated with fitting SLs in DED patients, including increased midday fogging, poor wettability, and subjective patient satisfaction, will also be presented, as well as a discussion on the key considerations for SL fitting in this population. Overall, while more research is needed to support the use of SLs in DED patients without associated corneal irregularities and other forms of OSD, the use of these lenses may prove to have a potentially wider role given their reported ancillary benefits in these populations.

## 1. Introduction

Scleral lenses (SLs) are large-diameter rigid contact lenses that vault over the cornea and land on the conjunctiva, overlying the sclera [1]. Traditionally, SLs are used for visual rehabilitation of patients with irregular corneas [2,3,4], such as, but not limited to, primary and secondary ectatic diseases, corneal scarring, post-refractive surgery, and post-penetrating keratoplasty. SLs have also been increasingly used for cases with regular corneas [2,3,4], including dry eye disease (DED) management and high refractive error correction.

DED is defined by TFOS DEWS II as “[…] a multifactorial disease of the ocular surface characterized by a loss of homeostasis of the tear film, and accompanied by ocular symptoms, in which tear film instability and hyperosmolarity, ocular surface inflammation and damage, and neurosensory abnormalities play etiological roles [5]”. SLs are applied with the space between the cornea and the lens filled with non-preserved saline. Thus, during SL wear, the cornea is always enclosed in a moist environment made of non-preserved saline and tears with no direct fluid evaporation [6]. Currently, in the treatment hierarchy of DED treatment by the TFOS DEWS II Report, SLs are placed at Step 3 out of the four steps, at a later point of consideration after other advanced interventions, such as overnight moisture chamber devices, intense pulsed light therapy, and topical and oral prescription medications, are to be considered [7]. Clinically, individual practitioners have anecdotally reported successfully managing many patients with DED with SL wear. Therefore, there have been discussions in the specialty contact lens community on whether SLs should be used earlier in DED management [8]. This article will review the existing evidence on the efficacy of SLs in managing DED, as well as discuss the potential advantages and challenges of fitting SLs in patients with DED.

## 2. Current Evidence Supporting the Use of SLs in DED and OSD Patients

### 2.1. SLs for DED Management with Associated Corneal Irregularities and Other Forms of OSD

Ocular surface disease (OSD) is an umbrella term that includes different conditions that impact the ocular surface [9], including DED. Many OSDs, such as exposure keratopathy and graft-versus-host disease (GVHD), can cause DED, or similar signs and symptoms [5,9]. SLs are often prescribed for patients with OSDs for ocular surface protection, corneal healing, and ocular symptomatic relief [10,11]. As severe OSD can often result in corneal irregularity, SLs can also effectively rehabilitate vision in these cases [12,13]. The efficacy of SLs in the management of OSD with associated corneal irregularities, including GVHD, Stevens–Johnson syndrome (SJS), Sjögren’s syndrome, exposure keratopathy, and post-refractive surgery dry eye, has been covered in previous review papers [10,11]. Overall, most reports have suggested the efficacy of SLs in managing OSD with studies of small sample size (level 2 evidence [7]), retrospective chart reviews (level 2 evidence [7]), or individual case reports (level 3 evidence [7]) [12]. Several studies have also included different types of OSDs in the study design or chart review, with only part of the study population consisting of pure DED patients [14,15,16]. In three studies fitting DED patients with SLs, significantly improved Ocular Surface Disease Index (OSDI) scores were observed [14,15,16]. In addition, a significant decrease in tear osmolarity [14] and improvement in corneal and conjunctival staining were reported [15], suggesting the potential utility of SLs in patients with only DED. In ocular GVHD, dry eye is reported to be the most common symptom [17]. Studies on SL management of ocular GVHD reported symptomatic relief of ocular dryness, irritation, and foreign body sensation with their use [6,18,19,20,21,22]. One study also showed significant improvement in OSDI scores post-SL fitting [20]. DED is estimated to affect one third of SJS patients in the chronic stage [23]. A retrospective chart review of 39 patients with SJS or toxic epidermal necrolysis (TEN) reported significant improvement in OSDI scores with SL wear [24]. Another case report also showed symptomatic relief of DED symptoms in one SJS patient with SL wear [25]. In exposure keratopathy, the exposure of the ocular surface leads to symptomatic DED in patients. SL wear significantly improved OSDI scores and corneal staining in a retrospective chart review of 29 patients with exposure keratopathy [26]. The prevalence of post-refractive surgery dry eye has been reported to be 36–75% [27]. Marty et al. used SLs for visual rehabilitation in 29 patients with post-refractive surgery ectasia and found concurrent significant improvement in OSDI scores with SL wear [28]. 

### 2.2. SLs for DED Management without Associated Corneal Irregularities and Other Forms of OSD

To investigate the available evidence on using SLs to manage DED even in the absence of corneal irregularities or other forms of OSD, a literature review was performed on PubMed on 15 February 2024, using the following keywords in combination: scleral lenses and dry eye disease. It resulted in 83 papers identified. After eliminating studies that strictly include non-DED OSD with associated corneal irregularities, no peer-reviewed papers and only two published abstracts remained, highlighting the paucity of evidence of this intervention in this patient population. One of the abstracts identified from the 2024 Global Specialty Lens Symposium consisted of a prospective, randomized, double-masked study that recruited 20 symptomatic soft-lens wearers without corneal irregularities [29]. Subjects completed a dry eye questionnaire (CLDEQ-8) and rated their end-of-day contact lens comfort and dryness with habitual soft contact lenses and SLs after 1 month of wear [29]. When compared to habitual soft contact lens wear, the results showed significant improvement in CLDEQ-8 score and subjective ratings of end-of-day contact lens comfort and dryness with SLs wear [29]. At the end of the study, 45% of the subjects indicated that they would like to continue wearing SLs and requested the SL parameters to be shared with their eye-care professional [29]. The authors concluded that switching symptomatic soft-lens wearers to SLs improved comfort and reduced dryness symptoms after 1 month of wear [29]. The other abstract from the 2024 Netherlands Contact Lens Congress by the same authors recruited 18 symptomatic non-contact lens wearers without corneal irregularities [30]. When compared to the baseline, significant improvement in CLDEQ-8 score and subject ratings of end-of-day contact lens comfort and dryness were found with SL wear [30]. Similarly to symptomatic contact lens wearers, at the end of the study, 44% of the subjects who were habitual non-contact lens wearers indicated that they would like to continue wearing SLs and requested the SL parameters to be shared with their eye-care professional [30]. The authors concluded that SLs improved comfort and reduced dryness symptoms after 1 month of wear in non-contact lens wearers who are symptomatic for mild to severe DED, and SLs should be considered as an option for management in this patient population [30]. 

These two recent abstracts are the only level 2 evidence available in the literature on managing symptomatic DED in the absence of corneal irregularities and other forms of OSD with SLs, suggesting that there is currently insufficient high-quality evidence to recommend the use of SLs in mild to moderate DED. The results of published studies on the efficacy of SLs in the management of DED with and without associated corneal irregularities and other forms of OSD have been summarized in Table 1.

## 3. Advantages of Fitting SLs in DED

Despite the lack of evidence, SLs are thought to be able to afford several advantages for managing symptomatic DED without corneal irregularities and other forms of OSD, especially when the patient is a symptomatic soft contact lens wearer. These advantages include ocular surface protection, corneal healing, elimination of contact lens dehydration and tear film evaporation, and improved visual acuity and its associated effect on wear comfort. 

### 3.1. Elimination of Tear Film Evaporation

Since the ocular surface is always enclosed in a moist environment with SL wear, there is no direct fluid evaporation. The natural tear film is thought to be made of a lipid layer and a mucoaqueous layer and contains numerous lipids, electrolytes, mucins, proteins, and metabolites [31,32]. The outer lipid layer is thought to prevent the evaporation of the aqueous layer, and it is shown to be thinned with soft contact lens wear [33,34]. This disruption of the natural tear film structure may lead to contact lens-related discomfort. SL wear, however, has one particular advantage compared to other lens types in this regard. The post-lens tear film of a SL consists of non-preserved saline and components of the natural tear film, resulting in a completely different structure with minimal tear exchange and evaporation [32]. Future studies involving detailed tear film analysis with SLs may help explain how SLs prevent or improve DED symptoms.

DED has been classified into two etiological categories, aqueous deficient dry eye (ADDE) and evaporative dry eye (EDE) [5]. The TFOS DEWS II Report states that the two DED types exist in a continuum with a preponderance of EDE in the population [5]. Furthermore, some studies have shown increased tear evaporation with soft contact lens wear [35,36,37,38], and one study reported increased report of DED symptoms concurrent with increased tear evaporation [37], supporting the hypothesis that increased tear evaporation plays a role in contact lens-induced DED. By keeping a stable post-lens tear film composed of tears and non-preserved saline, SL wear ensures that there is adequate hydration and no tear evaporation over the ocular surface. Therefore, both forms of DED patients, ADDE and EDE, may potentially benefit from SL wear. 

### 3.2. Elimination of Contact Lens Dehydration

Scleral lenses are not hydrated; thus, there is no contact lens dehydration associated with their wear. Soft contact lenses are prone to dehydration when worn, which depends on several factors, such as the material, humidity, air flow, and temperature [39,40]. It has been proposed that soft contact lens dehydration is associated with subjective reports of discomfort and dryness; however, a causative link has not been established [39,41,42,43,44]. Since SLs are made of rigid gas-permeable (GP) material with no water content, there is no contact lens dehydration, eliminating this as a potential factor in creating symptoms. It is worth noting that corneal GPs, although made of the same rigid material, are generally not considered a good option for DED management. Unlike SLs, small corneal GPs make direct contact with the corneal epithelium with blinking [45]. This mechanical stimulation of the corneal epithelial cells was hypothesized to underly the subjective complaint of foreign body sensation and discomfort with corneal GP wear [45]. Furthermore, compared to SLs, corneal GPs do not provide a stable post-lens tear film; rather, similar to soft contact lenses, corneal GPs may disrupt the composition of the tear film by thinning the lipid layer, potentially leading to increased tear evaporation [45,46]. 

### 3.3. Corneal Healing

**Figure 1:**
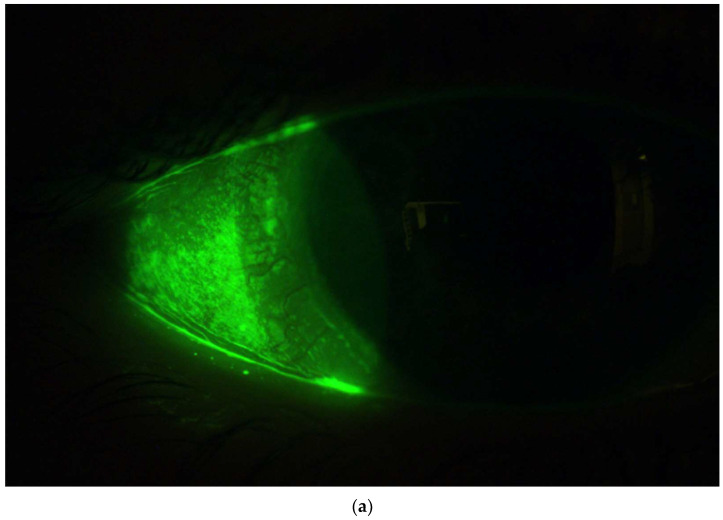

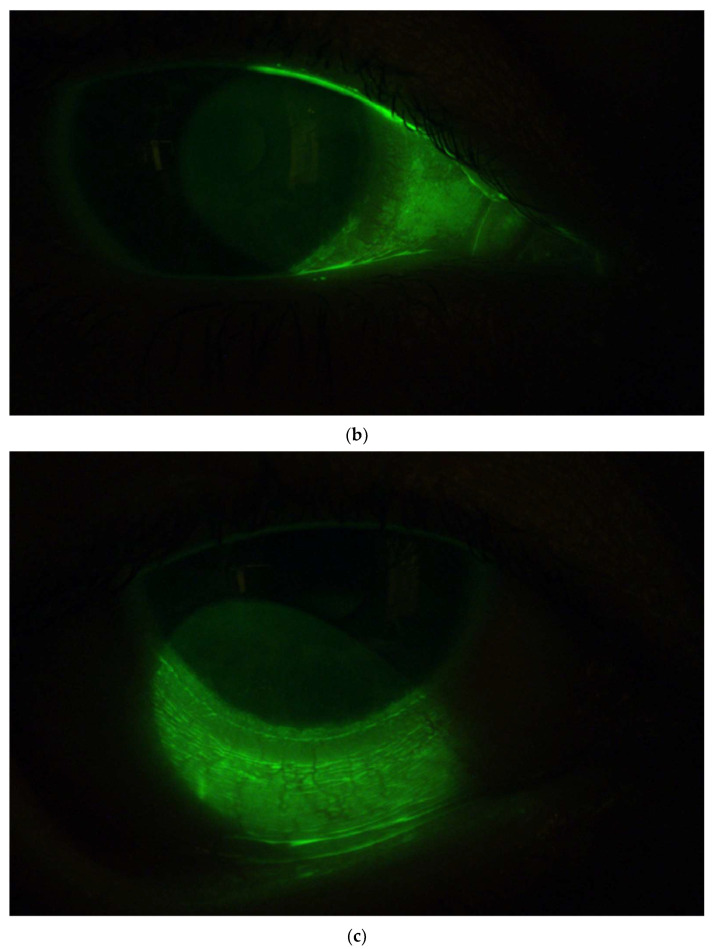
Images after removing a scleral lens in a symptomatic dry eye disease patient with regular cornea, who had corneal and conjunctival staining at the baseline visit. After wearing the lens for approximately 8 h, images with fluorescein were taken upon its removal. It reveals an absence of staining beneath the scleral lens. However, conjunctival staining was observed outside the edge of the scleral lens in the temporal (**a**), nasal (**b**), and inferior (**c**) quadrants of the still-exposed ocular surface area. Image credit Daddi Fadel.

SLs can potentially promote corneal healing and improve corneal health. SL wear reduces corneal staining according to several case reports and case series in OSD management [47,48]. A global survey of SL practitioners involving nearly 300 responses reported that the incidence of corneal staining reduced from 55% pre-SL wear to 35% post-SL wear [49]. The corneal healing effect of SLs is especially well documented in the management of neurotrophic keratitis, where SL wear can help heal persistent epithelial defects, according to multiple case reports and case series [50,51,52,53]. The exact physiological pathway of corneal healing through SL wear is unknown. Although no study has been conducted to investigate the effect of SL wear on corneal staining in DED cases, reduced corneal staining has been observed clinically after SL wear (Figure 1) and would be hypothesized to occur more broadly if these lenses were used. 

### 3.4. Improved Visual Acuity

SLs can potentially also improve visual acuity and the associated comfort of contact lens wear. Research on soft contact lenses has shown that reduced visual acuity is associated with worse perceived contact lens wear comfort [54,55]. SLs have been found to provide improved best-corrected visual acuity than habitual visual correction (soft contact lenses or glasses) in eyes with regular corneas [49,56]. The post-lens tear film behind a SL can mask anterior corneal astigmatism and other irregularities [57], providing a smoother refracting surface that may lead to better visual acuity. Therefore, the best-corrected visual acuity improvement with SL wear may contribute to better wear comfort. However, to date, no study has been conducted on this subject with SLs, so whether this translates to SLs is unknown. 

Overall, based on research with soft contact lenses, inferences on the advantages of wearing SLs based on DED patients without associated corneal irregularities or other forms of OSD can be made to support prescribing SLs in this patient population; however, more SL-specific research is needed. As the above-proposed advantages of SL wear directly address issues of contact lens discomfort related to soft contact lens wear, future studies should aim to include symptomatic soft contact lens wearers and non-contact lens wearers when investigating the efficacy of SLs when managing DED, as shown by the two published abstracts [29,30].

## 4. Challenges of Fitting SLs in DED

Despite the potential benefits of SL fitting in DED patients with regular corneas, several challenges must be considered when fitting SLs in this population, including midday fogging (MDF), wettability, and patient expectations.

### 4.1. MDF

**Figure 2:**
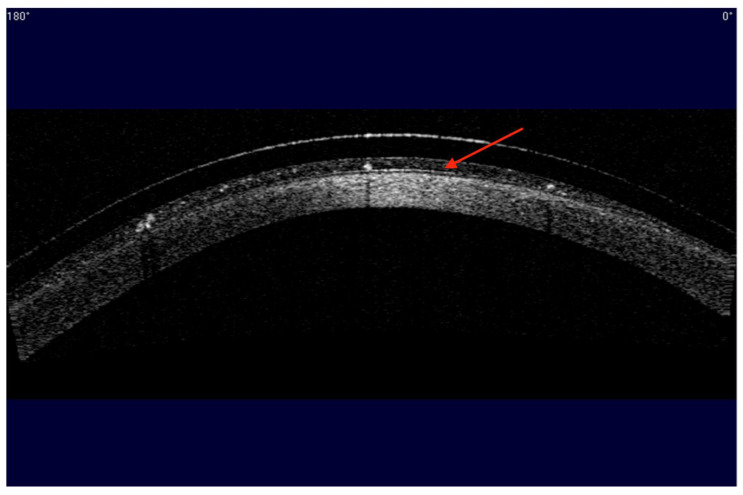
Anterior segment OCT image of midday fogging in a scleral lens patient. Debris of different sizes accumulate in the post-lens tear film, giving the post-lens tear film (red arrow) an opaque appearance. Image credit Sharon Qiu.

MDF is a common complication estimated to affect between 26% to 46% of SL wearers [58,59,60]. In MDF, debris is trapped in and accumulates in the post-lens tear film created by a SL (Figure 2). With a significant accumulation of debris, visual acuity can be impacted [58]. According to a recent survey, patients with DED have a higher incidence of MDF, with 50 out of 69 patients who wore SLs for DED reporting MDF [61]. This is a 75% rate of MDF in this DED population of SL wearers who experience MDF, a much higher incidence than the 26% to 46% average reported in other SL studies [58,59,60]. Another study reported a relationship between the OSDI score of SL wearers and MDF, with participants reporting MDF having a more severe OSDI compared to those who were MDF-free [62]. The higher incidence of MDF could be due to inflammation, a potential common denominator for DED and MDF. The TFOS DEWS II definition included “ocular surface inflammation” as one of the underlying etiologies of DED [5]. 

Although the exact composition of the post-lens tear film is not well understood, increased levels of inflammatory markers have been shown to be linked to increased incidence of MDF [32,63]. Leukocytes, particularly neutrophils, have been found in the post-lens tear film of SL patients, especially those who reported MDF [59]. Levels of inflammatory mediators, such as matrix metalloproteinase (MMP)-9 and -10, are also significantly elevated in the post-lens tear film after 8 h of SL wear when compared to the levels in basal tears [64]. No tear film analysis study has been performed on DED patients who report MDF to date. Although more evidence is needed, patients who wear SLs for DED may have an increased likelihood of experiencing MDF that would need to be proactively managed.

### 4.2. Poor Wettability

**Figure 3:**
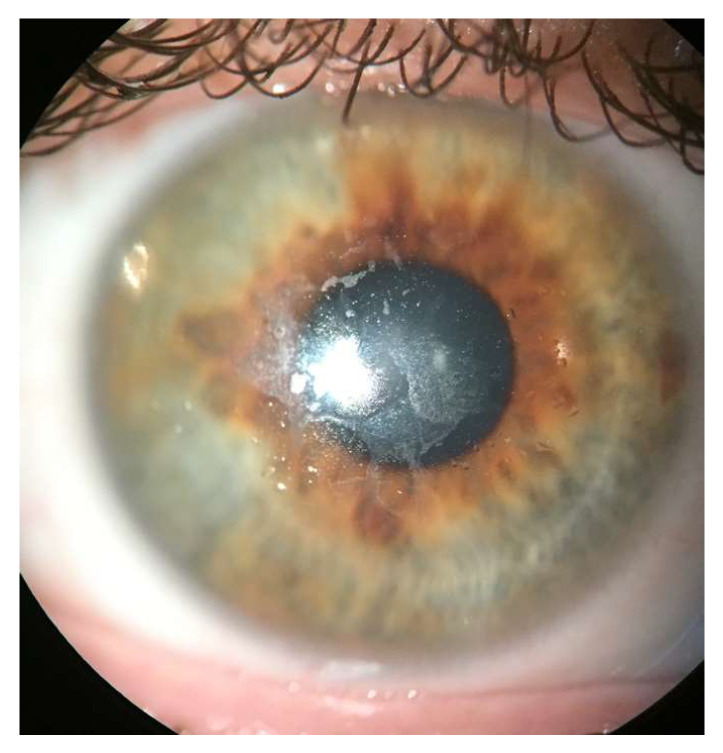
Poor wettability over a scleral lens as shown by a non-sharp light reflection on the lens surface. Image credit Daddi Fadel.

Another challenge of fitting SLs in the DED population is poor lens surface wettability. Contact lens wettability describes the ability of a liquid to spread onto and maintain contact with a surface [65]. Poor wettability indicates a non-stable tear film distribution over a contact lens surface [66] and is associated with transient blurred vision, contact lens intolerance, and symptoms of discomfort and dryness in the soft contact lens literature [67,68,69]. It is thought that in DED cases where meibomian gland dysfunction (MGD) is a factor that excess deposition of meibum onto the ocular surface can reduce wettability [70]. In the context of SLs, poor wettability would manifest as a non-sharp light reflection on the lens surface and often has a “greasy” appearance [71] (Figure 3). Although no studies have been undertaken to investigate the incidence of surface wetting issues in DED patients who are SL wearers, it has been observed clinically that patients with DED and other OSDs are more prone to suffer from poor wettability, which would suggest another barrier to successful SL implementation in this population [71].

### 4.3. Patient Expectations

It may also be more difficult to manage patient expectations when fitting DED patients with SLs. Many practitioners have anecdotally reported that DED patients without associated corneal irregularities or other forms of OSD often have a lower level of satisfaction with SL wear compared to patients who require SLs for visual rehabilitation. A study involving 178 patients reported that those with DED ranked comfort and overall satisfaction lower than patients with other conditions, such as keratoconus, penetrating keratoplasty, irregular astigmatism, and corneal dystrophy [72]. The authors commented that the lower score was partly due to MDF requiring reinsertion of SLs during the day [72]. A second reported factor contributing to lower patient satisfaction was the presence of deposits on the lens, which can be reduced by increasing the frequency of lens cleaning [73]. A third reason for dissatisfaction could be due to issues with the conjunctiva, which is not entirely covered by a SL. For example, conjunctival sensitivity [74] and conjunctivochalasis [75] were shown to be associated with DED signs and symptoms. A final reason may be reduced motivation to wear SLs when the visual benefits are not evident, especially during the initial fitting period [76]. In a study by Macedo-de-Araújo, a sample of majority soft contact lens wearers and non-contact lens wearers were fit into SLs [76]. At 12-month follow-up, more subjects with regular corneas dropped out of SL wear compared to subjects with irregular corneas, and the reason for dropout was primarily due to handling issues [76]. The authors hypothesized that because vision with SLs was on par with the habitual correction, subjects with regular corneas had less motivation to overcome issues associated with the learning curve of handling SLs for new wearers [76]. Although not specifically investigated in the study, this would be true for DED patients without associated corneal irregularities and other OSDs. More studies are needed on patient satisfaction and reasons for dissatisfaction in this particular patient population.

## 5. Specific Considerations of SL Fitting in DED Patients

The successful management of DED patients with SLs depends on several factors, including existing OSD management, rewetting drops, lens parameters, and filling solution considerations. 

When fitting DED patients with SLs, practitioners need to keep in mind that SLs can only alleviate the symptoms of dryness but will not treat the underlying causes of dryness. If the underlying cause of dryness is inflammation, such as in patients with MGD, blepharitis, or allergic conjunctivitis, therapeutic management of existing OSD needs to be initiated to treat the underlying inflammation before or concurrent with SL wear to ensure fitting success. 

No study to date has investigated the efficacy of rewetting drops over SLs. Clinically, some SL patients reported relief from residual dryness at the end of the day with rewetting drops. Similar to advice given to soft contact lens wearers, non-preserved and contact lens-compatible rewetting drops would be preferred over preserved and non-contact lens-compatible rewetting drops [77]. When the SL alone does not provide enough symptomatic relief for DED, if the patient is currently treated with autologous serum, some practitioners recommend mixing autologous serum with non-preserved saline in the post-lens tear film with anecdotal success. A case report discussed the resolution of persistent epithelial defects in a case of severe neurotrophic keratopathy by adding autologous serum to the SL fluid reservoir [78], but there is currently no other evidence in the literature to support the efficacy of this approach. 

When designing the initial SL, diameter is often the first parameter to be determined [79]. There is no consensus on what diameter works best for DED patients without associated corneal irregularities or other forms of OSD. On the one hand, fitting SLs less than 15 mm in diameter may be attractive since the cornea is regular, and small-diameter SLs are easier to fit for the novice practitioner and handle for the patient [80]. On the other hand, experienced practitioners may opt for larger-diameter SLs for the larger conjunctival coverage that may contribute to the overall sensation of dryness and reduced conjunctival staining, as shown in Figure 1.

Ensuring proper edge alignment is also critical for fitting success. A tight or loose edge may exacerbate ocular irritation in DED patients [81]. Discomfort may come from the pressure exerted by a tight-fitting lens edge or the friction between the eyelid and a loose-fitting lens edge [81]. Therefore, care should be taken to troubleshoot edge alignment problems, such as edge lift, conjunctival impingement, or conjunctival blanching, by assessing the settled SL edge on the eye. Furthermore, the observation of an indentation ring or conjunctival staining after lens removal may indicate a tight edge [71]; upon fluorescein instillation, the observation of sectorial fluorescein seepage into the post-lens tear film may indicate subtle edge lift [71]. If the SL design allows, toric, quadrant-specific, or free-form haptics should be added to ensure optimal alignment, as research on anterior segment shape has shown that only 5.7% of sclera were spherical [82].

To minimize MDF, besides managing existing OSD, lens parameters and filling solutions can both be optimized. Although studies were inconclusive, the general advice is to eliminate excessive fluid reservoir thickness (FRT) in any zone of a SL, including central, mid-peripheral, and limbal areas [63,71]. Excessive FRT provides the space for debris to accumulate and may induce additional inflammation on the ocular surface [63]. Edge alignment is also key, since a loose edge may allow the influx of debris, and a tight edge may worsen ocular surface inflammation [63]. When considering alternative filling solutions, a non-preserved filling solution that mimics the pH and ionic composition of natural tears may be used. A study in 22 SL wearers showed a non-statistically significant reduction in MDF grading on OCT images with this alternative filling solution [83]. Furthermore, the median OSDI scores and VAS scores for dryness, grittiness/foreign body sensation, burning/stinging, and overall pain/discomfort were significantly improved when this alternative solution was used [83]. Finally, some practitioners reported reduced MDF by adding a high-viscosity, non-preserved artificial tears in the post-lens tear film [81], although no study has been conducted to investigate the efficacy of this approach. 

If poor wettability is observed, the practitioner first needs to ensure that the issue is not due to laboratory defects or poor lens conditioning with a proper multipurpose solution before dispensing to the patient [84]. If poor wettability persists at follow-up appointments after careful lens inspection and proper soaking, practitioners should first review existing skin care products, hand soap, and make-up products with the patient to ensure that no excessive oily deposits are introduced onto the lens surface to impact wettability [71]. Practitioners should ensure that the proper lens material is chosen to provide a low contact angle and, therefore, good wettability [70]. Moreover, a polyethylene glycol (PEG)-based surface coating may be added to improve wettability [85]. In a study involving 21 SL wearers with DED, the application of the Tangible Hydra-Peg^TM^ (Tangible Science LLC, Menlo Park, CA, USA) surface coating was associated with statistically significant reduced lens discomfort, DED symptoms, corneal sodium fluorescence staining, tear break-up time, and frequency of MDF [85].

## 6. Conclusions

Despite the lack of evidence in the literature to support fitting SLs in DED patients without associated corneal irregularities or other forms of OSD, practitioners are increasingly offering SLs in this patient population due to the many potential benefits while keeping the challenges in mind. More well-designed studies demonstrating the benefit of SLs in DED patients without concurrent corneal diseases are needed before SLs can be considered in early-to-moderate DED management under the existing DED treatment hierarchy.

## Figures and Tables

**Table 1:** Studies on the efficacy of scleral lenses (SLs) in the management of dry eye disease (DED) with and without associated corneal irregularities and other forms of ocular surface disease (OSD). CLDEQ-8 = Contact Lens Dry Eye Questionnaire-8. GVHD = graft-versus-host disease. LASIK = laser in situ keratomileusis. NEI VFQ-25 = National Eye Institute 25-Item Visual Function Questionnaire. OSDI = Ocular Surface Disease Index. PED = persistent epithelial defect. PRK = photorefractive keratectomy. SJS = Stevens–Johnson syndrome. TEN = toxic epidermal necrolysis.

Paper/Abstract	Type of Study	Level of Evidence [7]	Disease(s)	Subject Information	Study Outcome	Additional Comments
Fadel et al., 2024 [29]	Prospective, randomized, double-masked study	2	DED without corneal irregularities or other OSDs	20 symptomatic soft-lens wearers	Significant improvement in CLDEQ-8 score and subjective ratings of end-of-day contact lens comfort and dryness when comparing SL wear to habitual soft contact lens wear at 1 month; 45% of subjects would like to continue wearing SLs	N/A
Wong et al., 2024 [30]	Prospective, randomized, double-masked study	2	DED without corneal irregularities or other OSDs	18 symptomatic non-contact lens wearers	Significant improvement in CLDEQ-8 score and subject ratings of end-of-day contact lens comfort and dryness when comparing SLs wear to baseline at 1 month; 44% of subjects would like to continue wearing SLs	Subjects had mild to severe DED prior to SL wear
La Porta Weber et al., 2016 [14]	Prospective interventional case series	2	SJS (22 eyes), Sjogren’s syndrome (11 eyes), ocular GVHD (2 eyes), DED post-LASIK (2 eyes), undifferentiated OSD (4 eyes)	25 subjects with different types of OSDs	Significant improvement in tear osmolarity values, van Bijsterveld scores, OSDI scores, and quality of life (via SF-36v2 questionnaires) when comparing 12 months after SL wear to baseline	N/A
Moon et al., 2021 [15]	Prospective, open-label and single-arm clinical trial	2	PED from various causes (10 eyes), chronic ocular GVHD (6 eyes), SJS (4 eyes), severe DED (1 eye)	13 subjects with intractable OSDs	Significant improvement in corneal and conjunctival staining, OSDI scores, and visual function (via NEI VFQ-25) when comparing 12 weeks after SL wear to baseline	N/A
Asghari et al., 2022 [16]	Retrospective chart review	2	DED (27 eyes), corneal ectasia (16 eyes), corneal ectasia + DED (9 eyes), cornea scar/opacity (8 eyes), limbal stem cell deficiency (7 eyes), SJS/TENS (4 eyes), exposure keratopathy (3 eyes), filamentary keratitis (2 eyes), atopic keratoconjunctivitis (2 eyes), corneal neuralgia (2 eyes), neurotrophic keratopathy (2 eyes), ocular GVHD + DED (2 eyes), pathological myopia (1 eye), PED (1 eye), post-penetrating keratoplasty (1 eye), other (8 eyes)	43 subjects with different types of OSDs and corneal irregularities	Significant improvement in OSDI scores when comparing follow-up (after 6 months of SL wear) to baseline	N/A
Bae et al., 2023 [18]	Retrospective single-center chart review	2	Ocular GVHD	9 subjects with chronic ocular GVHD	Significant improvement in subjective report of dry eye symptoms and quality of life at the time of interview compared to baseline	Median duration of wear at time of interview was 58 months (range 1–110)
Bligdon et al., 2021 [19]	Survey	2	Ocular GVHD	306 subjects registered with the Blood and Marrow Transplant Information Network	The most common symptom was gritty, dry eyes (87%). In current wearers of SLs (13% of respondents), SL wear improved symptoms of dryness/grittiness of the eyes (94%), eye pain (92%), and quality of life (89%)	56% of those wearing SLs wished the treatment was recommended sooner
Magro et al., 2017 [20]	Retrospective multi-center chart review	2	Ocular GVHD	60 subjects with chronic ocular GVHD	Significant improvement in OSDI scores and corneal staining (via Oxford scores) when comparing 2 months after SL wear to baseline	N/A
Schornack et al., 2008 [21]	Retrospective single-center chart review	2	Ocular GVHD	5 subjects with chronic ocular GVHD	Significant improvement in subjective report of comfort when comparing end of the follow-up period (4–14 months) to baseline	N/A
Takahide et al., 2007 [22]	Retrospective single-center chart review	2	Ocular GVHD	9 subjects with chronic ocular GVHD	Significant improvement in OSDI scores and subjective report of ocular symptoms when comparing end of the follow-up period (1–23 months after SL fitting) to baseline	N/A
Jacobs et al., 2007 [6]	Survey	2	Ocular GVHD	33 subjects with chronic ocular GVHD	Significant improvement in subjective report of ocular symptoms (pain, photophobia, and general quality of life) comparing time of the survey to baseline	SL fitting was completed between December 2002 and February 2005; survey was conducted between November 2004 and February 2005
Tougeron-Brousseau et al., 2009 [24]	Retrospective single-center chart review	2	SJS and TEN	39 subjects (67 eyes) with SJS and TEN	Significant improvement in OSDI scores and NEI VFQ-25 scores when comparing 6 months after SL wear to baseline	N/A
Fine et al., 2003 [25]	Case report	3	SJS	1 subject with SJS	Subjective improvement in symptoms of severe dry eye	N/A
Chahal et al., 2017 [26]	Retrospective single-center chart review	2	Exposure keratopathy	18 subjects with exposure keratopathy that completed SL fitting	Significant improvement in OSDI scores and corneal staining when comparing end of follow-up period (between 1 September 2009 to 30 June 2014) to baseline	N/A
Marty et al., 2022 [28]	Prospective study	2	Corneal irregularities post-refractive surgery—LASIK (24 eyes), PRK (4 eyes), mechanical keratomileusis (4 eyes), and radial keratotomy (3 eyes)	19 subjects with post-refractive surgery-related DED	Significant improvement in OSDI scores when comparing end of follow-up period (5–15 months after SL fitting) to baseline	N/A
